# Cholera Epidemic and Dysentery in Buffalo

**Published:** 1849-09

**Authors:** 


### EDITORIAL DEPARTMENT.

---

*Epidemic Cholera and Dysentery in Buffalo.*—The epidemic cholera has steadily declined since our last No. was issued. For the week ending Sept. 1st, the number of cases was 155: of deaths, 74. During the previous week there were reported 204 cases, and 82 deaths. All the cases reported up to the 1st inst., since the commencement of the epidemic, amount to 2505; of deaths, 858. The epidemic at this date (Sept. 5th) may be considered to have ceased. Three deaths were reported during the twenty-four hours ending yesterday noon, and one death only for the twenty-four hours ending at noon this day. We hope soon to have leisure to prepare some account of the disease as it has presented itself in this city, and to analyze the cases that have passed under our observation, the records of which have been preserved.

Dysentery has prevailed to some extent in this city for the past few weeks, presenting the characters belonging to the epidemic form of the disease. In some cases it has evinced considerable malignancy. So far as our observation and knowledge of it has extended, torimna and tenesmus are not prominent symptoms. The mucous tunic of the entire large intestine appears to be involed, and, also, to some extent, the small intestine. This opinion is predicated upon the phenomena during life wholly, opportunities for autopsical examination not having as yet been presented.

In severe cases the pulse becomes frequent, accompanied by prostration, and, in some instances, typho-mania. The discharges are apt so be sanguinolent, and a strong hæmorrhagic tendency distinguishes some cases.

We have been informed that in other places a similar form of dysenteric disease has attended the decline of epidemic cholera, the latter affection appearing to become merged in the former.

In the city of New York epidemic cholera has greatly declined. At Rochester and Albany a few cases are reported daily. No cases have, as yet, been reported at Utica. It appears to linger at Cincinnati. It also prevails somewhat at Boston and vicinity, notwithstanding the bulletin of the Mayor, issued some months ago, giving assurance that the disease was not to be expected in that neighborhood.

